# Bibliometric Analysis on Tuberculosis and Tuberculosis-Related Research Trends in Africa: A Decade-Long Study

**DOI:** 10.3390/antibiotics10040423

**Published:** 2021-04-12

**Authors:** Aboi Igwaran, Chiedu Epiphany Edoamodu

**Affiliations:** School of Biological Sciences, University of Fort Hare, Alice 5700, South Africa; cedoamodu@gmail.com

**Keywords:** bibliometric study, collaboration, infection, *Mycobacterium tuberculosis*

## Abstract

Tuberculosis is one of the oldest known diseases and the leading communicable cause of deaths worldwide. Although several studies have been carried out on tuberculosis, no research has examined the publication trends in this area. Hence, this study aimed to fill the gap by conducting a bibliometric study in publications trends on tuberculosis and tuberculosis-related studies in Africa from 2010–2019 and explore the hotspots. Information in published documents on tuberculosis and its related studies from 2010 to 2019 were retrieved from the Web of Science (WoS) database. The bibliometric tool biblioshiny and Microsoft Excel 2016 were used to analyse the top leading journals, top cited documents, authors’ country production, country collaboration networks, most relevant authors, authors’ impacts, most relevant authors by corresponding author, most cited countries, university collaborations, most relevant affiliations, conceptual structural maps, title word co-occurrence networks, collaboration and significance of individual sources, university, country and keyword relations. A total of 3945 published documents were retrieved. The analyses showed that *European Respiratory Journal* was the leading journal in publications on tuberculosis studies with a total of 452 published articles, the WHO 2012 report was the most cited document with 2485 total citations while South Africa was the most productive country in tuberculosis publications as well as the leading country with the highest co-authorship collaboration. Analysis of top relevant authors revealed that Anonymous (133) and Dheda (44) were the two topmost relevant authors of tuberculosis publications, South Africa was the most relevant country by corresponding authors and the topmost cited country for tuberculosis publications. Furthermore, analysis of the university collaborations network showed that the University of Cape Town was the topmost university in Africa with the highest collaboration network, tuberculosis as a word had the highest co-occurrence network while the Three Field Plot diagram revealed the relations between universities, keywords and countries. This study provides a quantitative and qualitative analyses of the leading journals, most cited published articles, title word occurrences, and most relevant authors in published documents on tuberculosis and tuberculosis related studies from 2010–2019.

## 1. Introduction

Globally, the health of about 10.4 million persons is impacted yearly by tuberculosis (TB) resulting in approximately 1.7 million TB-related deaths with the majority of the deaths occurring in resource-limited nations [1]. TB is caused by *Mycobacterium tuberculosis* complex (MTBC) and it remains one of the global leading public health problems [2]. The genus *Mycobacterium* comprises of over 140 species [3] that are divided into three major group including MTBC, *M. leprae* and nontuberculous mycobacteria (NTM) other than MTBC and *M. leprae* [4]. Members of the MTBC includes *M*. *africanum*, *M*. *tuberculosis*, *M*. *pinnipedii*, *M*. *bovis*, *M*. *canettii*, *M*. *caprae*, *M*. *microti*, *M*. *bovis* BCG vaccine strain and *M*. *mungi* infecting humans as well as animals [5,6]. Of all these members of the MTBC, *M. tuberculosis* is an obligate human pathogen [2] and is the most prominent member of the MTBC causing TB in humans [7]. TB is one of the oldest known diseases and the leading communicable or infectious cause of global deaths [8]. In 2018, 7 million people were diagnosed and treated for TB [9] and out of which 1.5 million persons died due to this bacterial infection [10].

The main risk factors for tuberculosis include human immunodeficiency virus (HIV), older age, lower socioeconomic status such as homelessness, crowding, poor nutrition etc. [11]. Other risk factors connected with the development of tuberculosis in human include contact with TB patients, alcohol consumption and smoking [12]. Globally, efforts have been made to prevent or control TB, but this disease continues to be a significant public health concern in many countries due to the development of multidrug-resistant *M. tuberculosis* MDR-MTB. MDR-MTB occurs when these bacteria become resistant to the drugs or agents used in the treatment of the disease; and this is a serious problem that is increasingly becoming prevalent worldwide [13]. Drug resistance MTB or MDR-MTB is one of the main causes of deaths worldwide [14]. In addition to the burden caused by MDR-MTB, TB is the leading cause of death from a single infectious agent and the ninth leading cause of global deaths [15,16].

TB is a major communicable disease with a global prevalence and about one-third of the worldwide population is infected with *M. tuberculosis* [17]. Among those infected with TB, 9.3 million individuals develop active TB [17], and the distribution of TB epidemics varies by country, with the most prevalence or incidence being in Africa (72%), followed by India (27%), China (9%), Indonesia (8%), and Philippines (6%) [18]. In Africa, over 25% of TB deaths occur [9]. In 2018, the rate of TB in Africa was 275 (238–314) per 100,000 [19], and among the 30 most worldwide TB affected countries, four countries are in Southern Africa, six countries are in East Africa, three countries are in Central Africa and three countries are in West Africa countries [19]. TB poses a major challenge in Africa and is the leading cause of loss of healthy life years and the number one killer of persons living with HIV [14]. Hence, studies on tuberculosis or tuberculosis-related studies are important; but to the author’s knowledge, no bibliometric study has been carried out to assessed publication trends on tuberculosis and tuberculosis related studies over time in Africa. Furthermore, the number of research publications is increasing at a fast pace and it is becoming gradually impossible to remain current with everything that is being published. Likewise, the emphasis on experimental contributions has resulted in ample and disjointed research outputs [20] and the copiousness of disjointed research obstructs the ability to amass knowledge and actively collect information via a set of preceding published research articles [20]. Hence, literature reviews are gradually assuming a vital role in fusing past research findings to successfully use the existing knowledge base to advance a line of research and provide evidence-based understanding or insight into the act or practice to carry out a sustaining professional judgment [21].

Moreover, numerous studies have been carried out on tuberculosis worldwide based on the infection incidence. Studies from such research have to help obtain data or information about the up-to-date state of research, measure current practice, and find gaps. To allows researchers to ascertain and commence new lines of research and help policymakers to evaluate and suggest approaches to improve on the research gaps, a method of statistical analysis called bibliometrics is used to measure or assess the major developmental trends and the characteristics of a given research subject based on published research [22]. Bibliometric analysis is a tool use for obtaining data or information about scientific activity in a certain field of study. This tool uses publication patterns in the study areas using quantitative investigation and statistics to analyze citation data [23], summarize, visualize, and characterize a set or group of publications. The resulting data or information obtained from the visualizations or maps can be used to study the history of scientific research outputs in a particular or specific field and identify the prospective future research directions and opportunities for collaboration [24]. To the best of our knowledge, this is the first bibliometric study on tuberculosis research trends in Africa from 2010–2019.

## 2. Methods

Published documents in the Web of Science Core Collection (WoS) on tuberculosis between 2010–2019 were mined. The WoS consists of a wide conglomerate of quality and high-impact scientific studies with more than 12 million articles for bibliometric analysis [25]. The WoS was chosen because of the numerous amounts of secondary information available for its indexed papers which offered numerous possibilities for bibliometric analysis [26], and the WoS is the most frequent database used for bibliometric study [27]. No database used for bibliometric study is considered superior as there are extensive variations in citations data in each database [28]. To avoid documents whose focus was not on TB, we carried out the search using the title (tt) field rather than the topic (tp) field. The tt field encompasses articles’ titles, abstracts, and keywords, (from authors and keywords plus) and keywords (from authors and keywords plus) [26]. The dataset retrieved from WoS database was processed and analyzed for bibliometric indicators using bibliometrix R-package in R-studio v.3.4.1 [21]. To retrieve the required data, the following steps were carried out: (a) the word “tuberculosis” was entered in the WoS search engine as title search; (b) the timespan was set from 2010–2019; (c) the documents were refined by limiting the search countries/regions to only Africa countries, (d) the retrieved data were exported via BibTex and finally; (e) biblioshiny and Microsoft Excel were used for graphical and data analyses. In this study, degree-centrality was used to measure or estimate a given node’s significance in a network [26,29]. This metric signifies the number of ties to a node. Each connected tie is defined by the number of co-occurrences between the two nodes. In the network’s collaboration, each node’s size is a distinctive or unique and increasing function of the degree; hence the term degree-centrality. The thickness of each matric between two nodes is a unique function of the weight, and the weight or size of the node is the number of collaborations between two nodes.

In summary:

*Degree_i_ = Σ_j ≠i_ a_i,j_* (A)

*Thickness_i,j_ = N° of Collaborations between i and j* (B)

The weight is “A” if there is a collaboration network connection between the nodes *i* and *j* and 0 otherwise. In general, there is said to be a collaboration network connection between two nodes if they share at least one co-occurrence, and this can be explained by considering country or university collaboration networks. The networks analysis was grouped into (a) top papers, source, and authors relations; (b) title, keyword, and keyword plus analysis; (c) scientific collaboration mapping. The title words, keywords, and co-keyword analyses are methods used to visualize and describe the arrangement or structure of scientific fields of a certain group of publications [30], and the level of scientific collaboration is determined or measured among others co-citation or citation analyses [31]. Hence, the country networks’ scientific collaboration, university networks, and title word co-occurrence networks were analyzed using a biblioshiny tool. Biblioshiny is also an appropriate tool used to present general statistics and relations or associations between the most significant scientific collaboration elements using the Three Fields Plot (TFP) [32]. Furthermore, top country authors and university relations were also established using the Three Fields Plot (TFP) of the biblioshiny package. The TEP tool is a tool that makes it possible to visualize the main items of three field selected (authors, country, and country) that display how they are connected using a Sankey diagram.

## 3. Result

### 3.1. General Information on the Retrieved Documents

A total of 3945 documents were retrieved from the WoS between 2010–2019, with average citations per documents of 4.512 and average citations per year per document of 0.7116. Articles (1395) are the most published document type, followed by meeting abstracts (1214), editorial material (311) and reviews (150), etc. The retrieved documents included 11,664 authors with 16,711 author appearances, 3.65 of collaboration index and 900 single-authored documents. Table 1 lists the general information of the retrieved published documents from the WoS data on tuberculosis and tuberculosis-related studies between 2010–2019.

### 3.2. Topmost Leading Journals

The 21 topmost leading journals in publications on tuberculosis with their number of published articles between 2010–2019 are shown in Table 2. The six most prolific journals out of the list are *European Respiratory Journal* (452), *American Journal of Respiratory and Critical Care Medicine* (216), *PLoS One* (164), *International Journal of Tuberculosis and Lung Disease* (105), *BMC Infectious Diseases* (81) and *African Medical Journal* (79).

### 3.3. Topmost Global Cited Documents on Tuberculosis

Table 3 shows the full details of the 21 topmost global cited documents on tuberculosis retrieved from the WoS database from 2010–2019. From the result of the analysis, the top five most cited document are the WHO 2012 Global Tuberculosis Report, with 2485 total citations (TCs) followed by the 2010 WHO Treatment of Tuberculosis Guidelines, with 634 TCs, Moeller [ref] with 151 TCs, Machingaidze 2011, with 124 TCs and Pooran 2013, with 115 TCs.

### 3.4. Author’s Country Production

**Figure 1:**
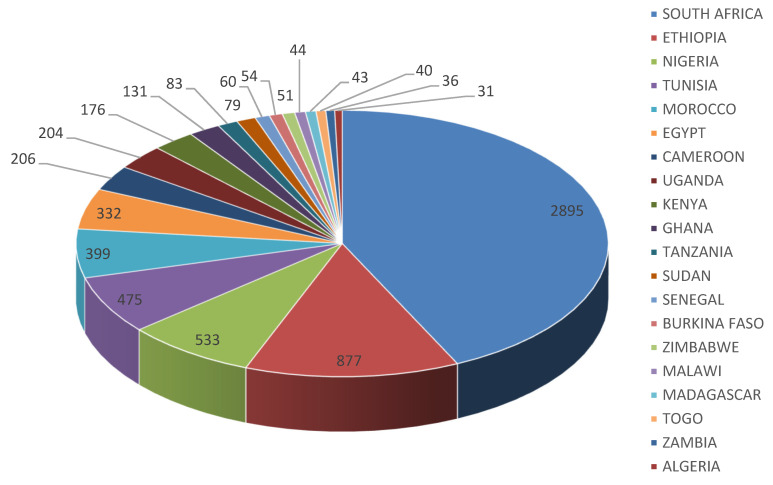
Authors’ country production in publications on tuberculosis.

Analysis of authors’ countries’ production outputs on tuberculosis and tuberculosis- related studies from 2010–2019 shows the top 20 most productive author countries in Africa on tuberculosis and tuberculosis-related studies. From the results, South Africa had the highest production output, with 2895 published documents on tuberculosis, followed by Ethiopia (877), Nigeria (533), Tunisia (475), Morocco (399). Figure 1 shows the top 20 Africa countries by their frequency of publications output on tuberculosis.

### 3.5. Analysis of Countries Collaboration Network

**Figure 2:**
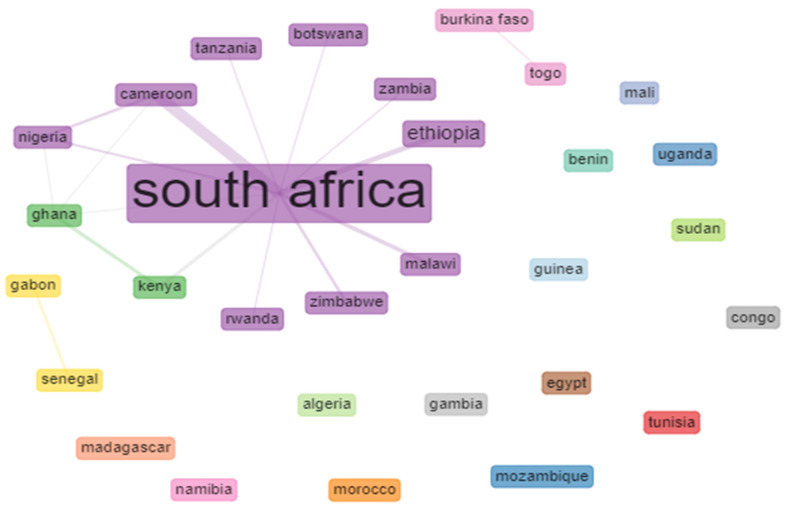
Co-authorship countries collaboration network on tuberculosis outputs.

The country collaboration and study hotspots can be provided with the visualization of the collaborations of co-authorship countries. The network analysis illustrates co-authorship co-occurrences between countries in tuberculosis publications. The countries’ collaboration networks on tuberculosis publications/outputs and its related studies from 2010–2019 show different clusters with pathways ranging from 0 to multiples. Each node signifies different Africa countries and each node’s diameter, or size denotes or represents the number of occurrence of authors country collaboration strength with other African countries, while the lines signify the collaboration networks or pathways between each country. In the clusters (Figure 2), South Africa had the highest number of collaborations as it collaborated with eleven (11) countries, followed by Ghana (*n* = 4 each), Nigeria and Cameroon (3) and Kenya, Burkina Faso, Gabon, and Senegal as each country collaborated with two other Africa countries. Most of the other African countries in Figure 2 had no collaboration network with other African countries. The thickness of the matric between two country denotes the country collaboration strength as the thickness of the matric between South Africa and Cameroon shows that these two countries have the highest collaboration network or strength.

### 3.6. Topmost Relevant Authors and Authors Impacts

Table 4 shows the top 21 most relevant authors in publications on tuberculosis-related research between 2010–2019 based on the number of published articles. The top 5 most relevant authors in the list are Anonymous (*n* = 133), Dheda (*n* = 44), Walzl (*n* = 27), Ameni (*n* = 25), Na and Van Helden (*n* = 24 each) and Goussard and Warren (*n* = 22 each). Based on the number of published articles, the five topmost authors impact on tuberculosis publication outputs include Dheda, with 44 publications with an h-index of 15 followed by Walzl (NP = 27; h-index = 11), Ameni (NP = 25; h-index = 10), Na and Van Helen (NP = 24; h-index = 5 and 9 respectively) and Goussard and Warren (NP = 22, h-index = 7 and 9 respectively). The authors’ full details of authors impact with their h-index, g-index, m-index, TC, number of publications (NP), and the publication year (PY) are as shown in Appendix A Table A1.

### 3.7. Top 22 Most Relevant Africa Countries by Corresponding Authors

Table 5 shows the details of the 22 topmost relevant African countries by corresponding authors on tuberculosis outputs based on the numbers of published articles (NPA), single country publications (SCP), multiple country publications (MCP) and frequency of their publications. The top seven African nations in the list were South Africa (NPA = 746; SCP = 717), Ethiopia (NPA = 223; SCP = 218), Tunisia (NPA = 153; SCP = 152), Morocco (NPA = 131; SCP = 131), Nigeria (NPA = 124, SCP = 122), Egypt (NPA = 95, SCP = 95) and Uganda (NPA = 48; SCP = 44) (Table 5).

### 3.8. Most Cited African Countries in Publications on Tuberculosis Studies

The top 20 most cited African countries in the retrieved documents from WoS database on tuberculosis related publications from 2010–2019 are shown in Table 6. The topmost cited countries are estimated using the institution or university affiliation of at least one author in each published article. The top five most cited African countries in the list are South Africa with a total citation (TC) of 7816 and average article citation (AAC) of 10.477) followed by Ethiopia with TC of 2125 with AAC of 9.529, Nigeria with TC of 555 with AAC of 4.476, Tunisia with TC of 514 with AAC of 3.359 and Uganda with TC of 446 and AAC of 9.292. Table 6 shows the top 20 most cited African countries in publications on tuberculosis studies.

### 3.9. Analysis of University Collaborations and Most Relevant Affiliations

**Figure 3:**
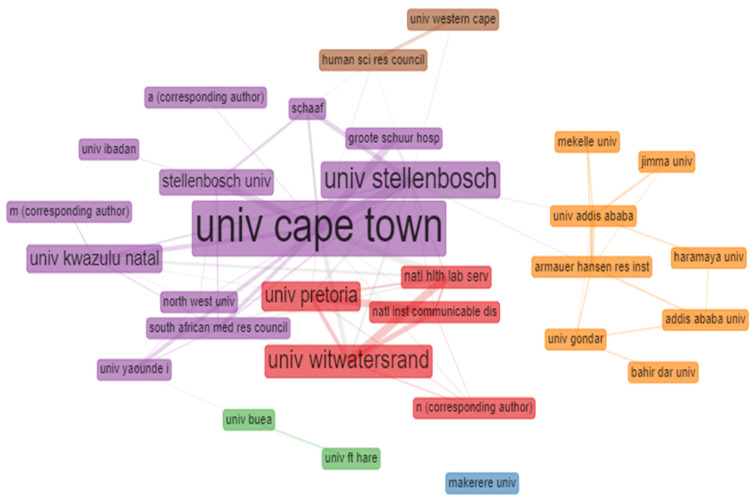
Co-authorship university collaboration networks on tuberculosis publications.

**Figure A1:**
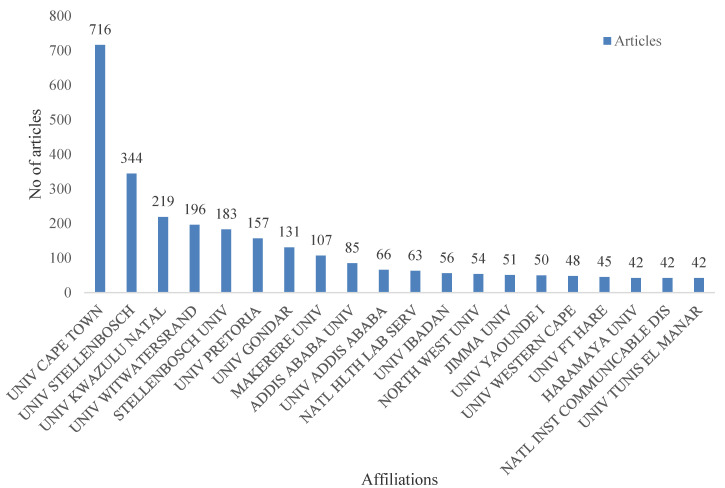
Most relevant institution affiliation on tuberculosis research outputs from 2010-2019.

Figure 3 shows the network collaboration of 30 African universities based on at least one author in each published document on tuberculosis studies. The results obtained from the analysis shows that the University of Cape Town had the highest collaboration network, followed by the University of Stellenbosch, University of Witwatersrand, University of Pretoria, University of Kwazulu Natal etc. while Makerere University has no collaboration network. Each node signifies the different universities and each node’s diameter denotes the university collaboration strength with other universities; the lines signify the collaboration networks or pathways between each university, while the thickness of the lines signifies the collaboration strength. Details of the top 20 most relevant universities in Africa based on the number of publications on tuberculosis between 2010–2019 is shown in Appendix A (Figure A1). The top six most relevant African universities or institutions with the highest collaborations networks on tuberculosis publications are the University of Cape Town (*n* = 716), University of Stellenbosch (*n* = 344), the University of Kwazulu Natal (*n* = 219), University of Witwatersrand (*n* = 198), University of Pretoria (*n* = 157) and University of Gondar, Ethiopia (*n* = 131) (Figure A1).

### 3.10. Analysis of Conceptual Structural Map

**Figure 4:**
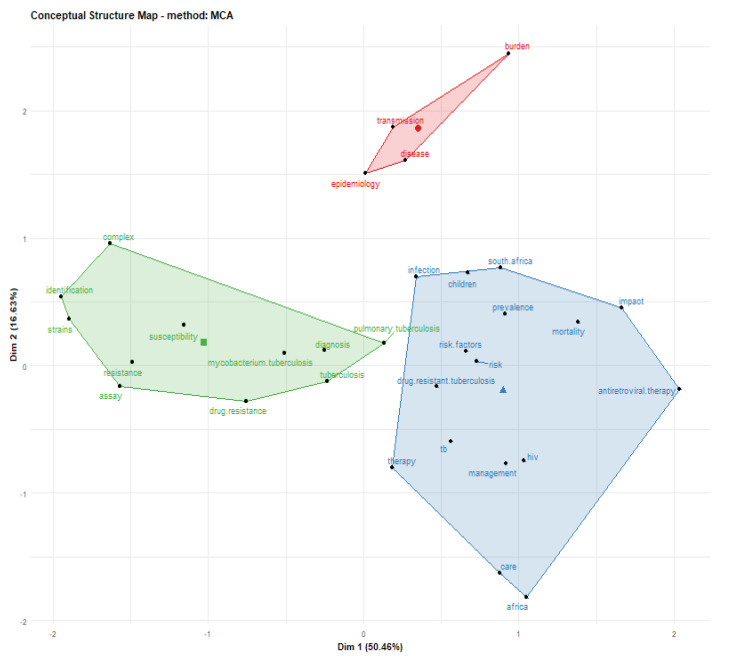
Share conceptual structural map in published documents on tuberculosis studies.

The conceptual structural map of publications on tuberculosis between 2010–2019 analyzed by factorial analysis of multiple correspondences in field keywords plus revealed three clusters of sizes 16, 11 and four, respectively, with components centered on drug resistance, burden, complex, mortality, drug resistance tuberculosis, children, risk factor, pulmonary tuberculosis, prevalence, identification, tuberculosis, HIV, *M*. *tuberculosis* etc. (Figure 4). The green cluster has *M*. *tuberculosis* as bacteria indicator, pulmonary tuberculosis as indicator of disease related term, the red cluster has disease as indicator of illness, the blue cluster has mortality as indicator of severity of infection, South Africa as indicator of country, HIV as indicator of co-infection and drug resistant tuberculosis as indicator of prevalent TB stains in this region.

### 3.11. Analyses of Title Words Co-Occurrence Network

**Figure 5:**
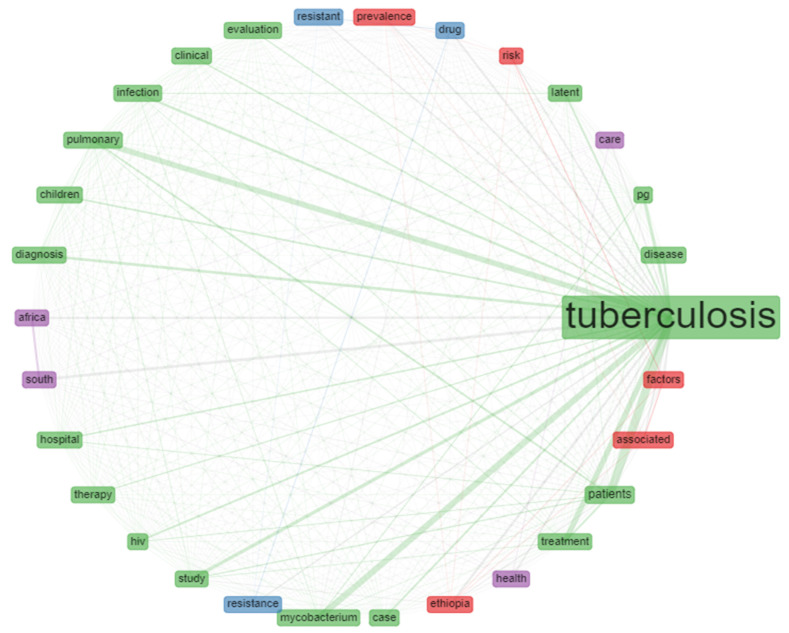
Title words co-occurrence network on tuberculosis.

The title words co-occurrence network was determined based on the number of occurrences, association, and co-occurrence of each title words in published documents on tuberculosis from 2010–2019. The title words co-occurrence network analysis, association, and node of 30 most frequently used words in published documents on tuberculosis studies from Africa is as shown in Figure 5. Each node signifies the title words while the lines denote the co-occurrence network or times each title word appear with other title words in published documents on tuberculosis. Each node’s size or diameter signifies the strength of occurrence of each title word in the published document on tuberculosis studies from Africa.

### 3.12. Analysis of Collaboration and Significance of Individual Sources

**Figure 6:**
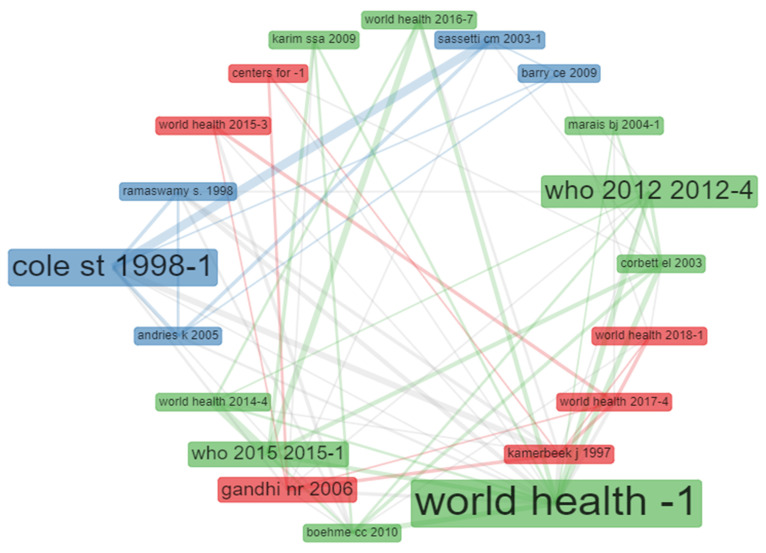
Co-citation network of sources in publications on tuberculosis studies (2010–2019).

The network collaboration of source-citations analysis covered the analysis of documents significance sources and co-citations of documents in the literature on tuberculosis studies. The document sources were journals, books, reviews, meeting abstracts, editorial materials, book chapters, etc. indexed in the WoS. Figure 6 represents the co-citation network sources in publications on tuberculosis studies. The rectangle’s size in the figure is based on the number of published documents. The more regular of the publications of a given document source cited the other publication source, the shorter the distance between the two document sources in the collaboration network analysis.

### 3.13. Top University, Country and Keyword Relations

**Figure 7:**
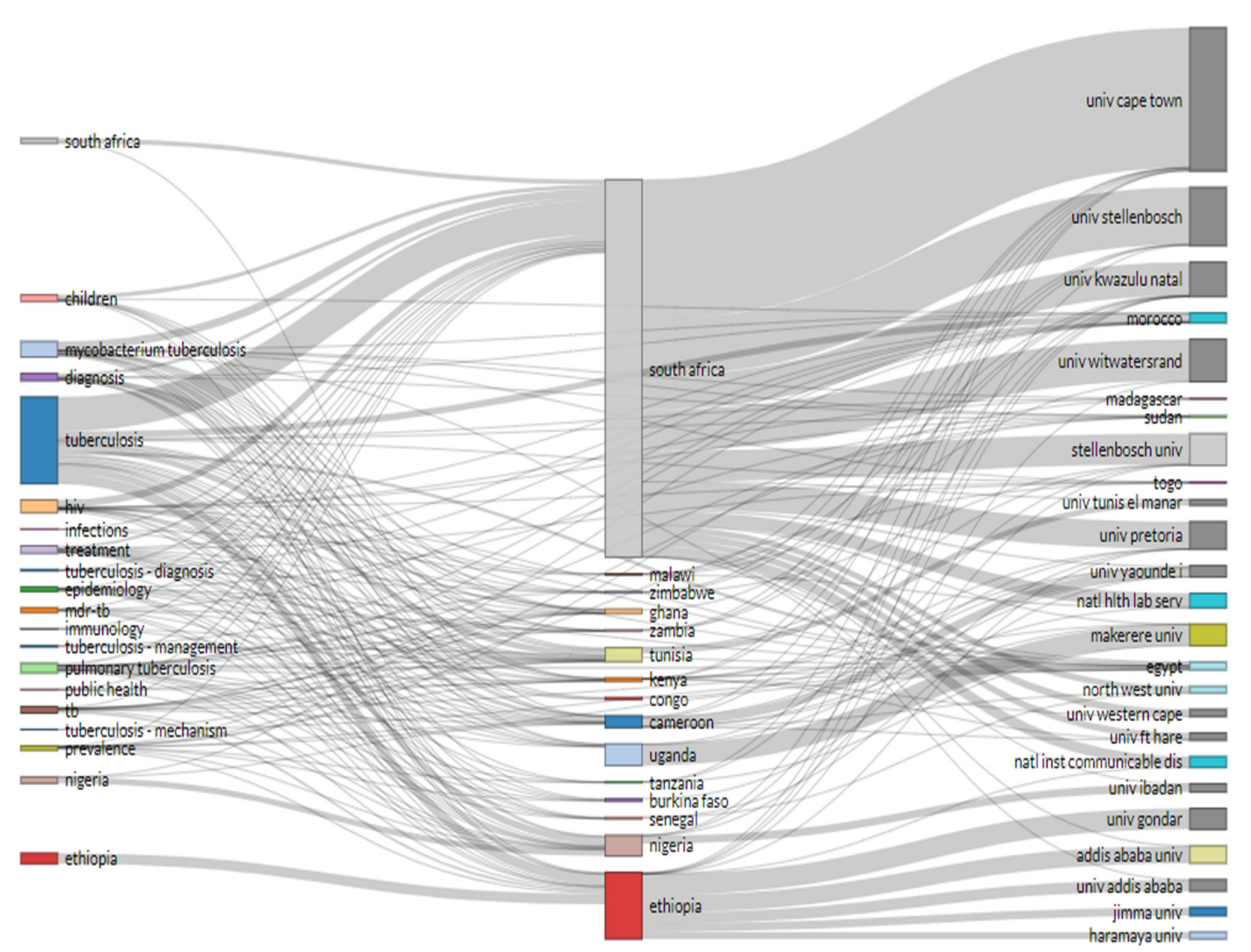
Relations between keywords (**left**), countries (**middle**) and affiliations (**right**) for research in tuberculosis literature.

The relations between universities, countries and keywords were visualized using the three fields plot (TFP). In this instance, the significant features were represented in the diagram by rectangles with a different colour. The height of the rectangles in the diagram of the TFP depended on the rate or value of the summation of the relations arising between the component of the rectangle represents (university, keywords, and countries) and the diagram of other elements. The more relations the component or element had, the higher the rectangle representing it. Figure 7 showed the TFP analysis of publications on tuberculosis centred on relations between the universities, keywords, and countries. The diagram demonstrated the top Africa university, keywords, and countries relations in publications on tuberculosis and its related studies.

## 4. Discussion

Bibliometrics analysis is a scientific study field that has attracted more attention among the scientific community [33]. Currently, bibliometric analysis or science mapping has advanced rapidly. This tool is applied to numerous research areas due to its effective way to assess the qualities of a particular journal or certain subject area [34]. In addition, bibliometrics is a discipline that deals with the widespread intersection and combination of statistics, mathematics, philology, and information science in a definite area [35]. It can also be used to analyse the progress of specific research trends which can be revealed by bibliometric indicators [36]. Bibliometrics analysis is important because of its distinctive advantages with its wide range of applications in various research fields of study such as sustainable energy [37], business and economics [38] and vague decision making [39]. This scientific tool or method have been used in several areas or field of study, including tropical medicine and citation analysis [40], publication patterns or trends on rational use of medicines in Iran [23] and research trends on periprosthetic joint infection [22].

This study analyzed publications trends on tuberculosis studies from Africa between 2010–2019. When studying the timeline for bibliometrics analysis, some vital factors need to be considered such as the inherent prejudice of bibliometrics against recently published articles or papers which might lead to some extremely vital papers not being involved or included in such analysis as it takes time to amass citations [41]. However, this bibliometric analysis was carried out to ascertain the research trends and gaps in published documents on tuberculosis studies from Africa between 2010 to 2019 and direct future study areas. From the analysis, a total of 3945 published documents were retrieved from the WoS database with 3.65 of collaboration index, 2567 of keyword plus and 3021 of author keywords. From the analysis, we observed that majority of the leading journals on tuberculosis and tuberculosis-related studies were published in high impact factor journals such as the *European Respiratory Journal* (*n* = 452), *American Journal of Respiratory and Critical Care Medicine* (*n* = 216) and *PLoS One* (*n* = 164). In the same vein, the topmost cited documents on tuberculosis or tuberculosis-related studies were also published in high impact factor journals and the total citations was between 64 and 634. The most cited document on tuberculosis retrieved from the WoS was the WHO title ‘Global tuberculosis report 2012′ [42] followed by the WHO 2010 title “Treatment of tuberculosis: guidelines, fourth edition”, Moeller et al.’s, publication in *Tuberculosis* [43] with 151 total citations, Machingaidze et al.’s paper, published in *Pediatric Infectious Disease Journal* [44] with 124 total citations, etc. (Table 3). The total number of document or journal citations and the impact factor of a journal are among the focal criteria used to define the scientific/academic impact of a paper [45], while the citation index is only accepted as an identifiability measurement [46].

As made evident by the statistical analysis, the total number of citations of topmost cited articles in this study is considered important because of the document’s quality. However, citation counts can vary due to the number of accumulated document or articles variable in diverse search engines [47]. The authors’ country production analysis showed the first 20 African country based on the number of authors country productions or publication outputs on tuberculosis and tuberculosis-related studies and the authors country production is between 31 and 2895. Among the top African countries in the list, South Africa had 2895 authors country production and was thus the highest country with the highest authors country production output on tuberculosis studies, and the lowest country in the list was Algeria with 31 authors country production (Figure 1). South Africa as a country having the highest number of author’s country productions could be linked with the country’s economy or research infrastructure and their numbers of publications/collaboration. The high rate of authors country production outputs focusing on tuberculosis in South Africa could also result from the high level of tuberculosis in the country, as South Africa is one six countries in the globe accounting for 60% of the worldwide tuberculosis burden [48,49]. Result from the analysis showed that Ethiopia is the second country in the list of country production outputs and Ethiopia is one of the 30 high TB; TB/HIV and MDR TB burden countries with an estimated TB incidence of 164 per 100,000 population of drug susceptible TB [15,50]. Likewise, analysis of country co-authorship network was also determined using bibliometrics, and this is according to the report of Durbach et al. [51]. Figure 2 is the diagram of the analysis of the collaboration network of the 30 most relevant African countries in publications on tuberculosis and tuberculosis- related studies. The result reveals that the highest country collaborations in publications on tuberculosis were between South Africa and Cameroon. Furthermore, most co-authors affiliated with South Africa institutions collaborated with other co-authors affiliated with other African countries like Tanzania, Botswana, Zambia, Ethiopia, Malawi, Zimbabwe, Rwanda, Kenya, Ghana, and Nigeria is as shown in Figure 2. According to Zhang et al. [52], the terms countries collaboration is used to describe documents that have been published via collaboration of authors from at least two different countries. Scientific network collaborations are the hallmark of existing academic research. Scientists are members of scientific collaboration networks looking for answers or solutions to scientific, economic, and social problems which regularly necessitate or require multidisciplinary approaches [53]. However, analyses of collaborative networks are mainly important in health innovation as a result of their complexity and multiple involvements of stakeholders and the increasingly reliant on interdisciplinary research [54]. From the result of the analysis of top relevant authors based on the number of publications on tuberculosis and tuberculosis-related studies there were 133 anonymous published documents retrieved from the WoS database and the least prolific authors were Hesseling, Ismail, Loot and Zar with 16 publications each on tuberculosis and tuberculosis-related studies. Table 5 shows the most relevant countries’ information by corresponding authors based on publications outputs on tuberculosis or tuberculosis related studies between 2010 to 2019. The result reveals the top 20 leading African countries on tuberculosis publications, and the top five African countries in the list were South Africa, Ethiopia, Tunisia, Morocco, and Nigeria with single country publications (SCP) of 717, 218, 152, 131, 122 and 95, respectively. From this result, South Africa had the highest SCP on tuberculosis publications among the 20 most relevant African countries by corresponding authors. Publication outputs or productivity are the numbers of scientific published articles or documents, which is a measure of a country’s scientific outputs [55]. The analysis of single country publications outputs on tuberculosis and it related studies is linked with authors or co-authors affiliation of country.

These high citations of the published document originated from these African countries shows the impact of tuberculosis on these scientific communities. This result is in line with the report of Merigo and Yang [56], as they state that the number of publications is linked or correlated to the output or productivity of the author while the number of document or article citations is correlated to its impact on the scientific community. If a country’s scientific output is measured carefully, it can be a useful indicator that can be used to describe a countries’ research activity [55]. Table 6 show the most cited African countries on tuberculosis or its related studies, and the topmost cited country in the list is South Africa with 7816 total citations and average article citations (AAC) number of 10.477, while the least country is Senegal with 25 total citations and an AAC of 1.786. Furthermore, the universities analysis shows that majorly of the university’s collaborations are in South Africa (Figure 3). From the result obtained from the universities collaborations analysis, the University of Cape Town had the highest collaboration strength or network, while Makereke University had no collaboration network with another African university. In this study, the term university collaboration was used to describe one author’s collaboration in an institution with another author in other institutions or universities. This was determined based on the number of published documents on tuberculosis through partnership. The lines signify collaboration networks between countries, and the thickness of each line signifies the amount or number of international co-collaborations. The result from the analysis reveals that majority of research societies have already formed a joined and tight collaboration network, particularly among the scientifically advanced countries, and this result is in line with the reports of Raan, [57] and Zhang et al. [52]. Co-citation is another way to analyse a research field and co-citation analysis that focused or concentrate on the conceptual structures [58]. The analysis of the conceptual structure map determined by K-means clustering and multiple correspondence analysis revealed three clusters (Figure 4). The clusters explained the types of infections, the severity of infection and strains of *Mycobacterium* species involved in causing tuberculosis. The green cluster has pulmonary tuberculosis as an indicator of infection site, tuberculosis as an indicator of the type of bacterial infection, the red cluster has epidemiology as the study indicator, transmission as an indicator of disease spread, and the blue cluster has HIV as an indicator of co-infection, Africa as an indicator of the continent, South Africa as an indicator of country and drug-resistant tuberculosis (DR-TB) as an indicator of transmission of DR-TB strains. This result is in line with the reports of Calver et al. [59] and Diarra et al. [60] as they reported hospital-acquired DR-TB strains in Africa.

Some of the major risk causes that have been linked with hospital-acquired DR-TB are lack of proper TB infection prevention and control measures, poor ventilation, delayed diagnosis, and overcrowded wards [61]. In nations with high HIV occurrence rate, over 75% of tuberculosis cases are HIV-associated [62] and the high rate of HIV prevalence rate in TB infected persons makes nosocomial spread or transmission a major driving force in the transmission of DR-TB strains [63]. Another method used to analyse a research field is the use of a co-word. The co-word analysis allows us to determine the main concepts explored by a field and to disclose and define the interactions between the diverse scientific research fields [64].

Furthermore, analysis of co-word is used to identify the associations between subjects in a research field and, consequently, help trace science development. Analysis of co-word also focused on identifying the hierarchies among the focused subject areas of a research problem [58]. The result of the title word and co-word co-occurrence network in publications on tuberculosis shows that the title word tuberculosis occurs most with *Mycobacterium* and pulmonary as the lines connecting these title words are thicker compared to other title words (Figure 4). The analysis of the conceptual structural map via co-word analysis showed three clusters of thematic networks. This result from this study’s is similar to the report of Galvão et al. [65] as they also reported three interrelated clusters of the conceptual structural map. Similarly, the analysis of individual and collaboration of significance sources in publications on tuberculosis shows that most of the documents were co-cited with WHO document while the report of Cole et al. [66] and Sassetti et al. [67] were the two most co-cited documents. Figure 7 presents the diagram from the analysis of three field plot on tuberculosis that centred on relations between the universities, countries and keywords. The analysis revealed that South Africa had more university affiliations in tuberculosis publications than other African countries in the list. The term tuberculosis analysis in the keyword indicates that all the countries in the diagram included the element as one of the keywords in their publications on tuberculosis or tuberculosis-related studies. Furthermore, publications affiliated to South Africa universities have strong relations with the term tuberculosis, HIV, MDR-TB and South Africa in their keywords.

## 5. Strengths and Limitations

One of this study’s major strengths is that it is the first and only bibliometric study to the best of our knowledge that characterizes published documents on tuberculosis in Africa from 2010–2019. By including all journals produced in the study region, we were able to generate a broad, comprehensive list of the top-cited journals, top-cited countries, top-cited documents, and most relevant countries by corresponding authors. There are also some limitations to the study. First, bibliometric investigation on newly published high-quality articles would be overlooked or ignored. This is a drawback connected to the effect of the number of times cited. Furthermore, the worth or value of an article influences and progress in a field or area cannot be known only by the number of citations. Furthermore, paper publication’s language plays a key role with favouritism or bias towards articles published in English language journals. In addition, low publication/collaboration rate of a country should not suggest that the quality of scientific research is low.

## 6. Conclusions

This study provides quantitative and qualitative analyses of the top leading journals, most cited published papers, most relevant authors in publications on tuberculosis from 2010–2019. This bibliometric study also provides insights into authors research outputs, countries collaborations, and relevant countries by corresponding authors. This will help create or generate evidence-based reports, evaluations, and visualizations of research outputs on tuberculosis research. Furthermore, we observed little or no collaboration between authors from non-English or less developed African countries with other English-speaking or developed African countries. In this setting, the involvement in collaboration of these countries with other African countries in research activity should be promoted, mostly those initiatives that consider their priorities and interests [68,69]. Therefore, the increase of united academic collaboration or partnerships among English and non-English speaking African countries may be an outstanding mechanism to achieve this goal. In addition, collaboration or partnerships among underdeveloped and developed African countries with high quality research infrastructure could help some of these African countries with low publication/collaboration rate to increase their numbers of publications/collaboration.

## Figures and Tables

**Table 1:** General information in retrieved published documents.

Description	Results
Sources (journals, books, etc.)	882
Documents	3945
Average years from publication	4.97
Average citations per documents	4.512
Average citations per year per doc	0.7116
Article	1395
Article; book chapter	41
Editorial material	311
Editorial material; book chapter	13
Meeting abstract	1214
Review	150
Keywords plus (ID)	2567
Author’s keywords (DE)	3021
Authors	11,664
Author Appearances	16,711
Authors of single-authored documents	536
Authors of multi-authored documents	11,128
Single-authored documents	900
Authors per document	2.96
Co-authors per document	4.24
Collaboration index	3.65

**Table 2:** Top 21 leading journal in tuberculosis documents.

Rank	Sources	Articles
1	*European Respiratory Journal*	452
2	*American Journal of Respiratory and Critical Care Medicine*	216
3	*PL* *oS ONE*	164
4	*International Journal of Tuberculosis and Lung Disease*	105
5	*Bmc Infectious Diseases*	81
6	*Pan African Medical Journal*	79
7	*Tropical Medicine & International Health*	77
8	*International Journal of Infectious Diseases*	75
9	*Lancet Infectious Diseases*	67
10	*Lancet*	60
10	*Revue De Pneumologie Clinique*	60
11	*Gastroenterology*	59
11	*Samj South African Medical Journal*	59
12	*BMC Public Health*	49
13	*Pneumologie*	48
14	*International Journal of Mycobacteriology*	46
15	*Lancet Respiratory Medicine*	44
16	*Egyptian Journal of Chest Diseases and Tuberculosis*	43
17	*Clinical Infectious Diseases*	33
18	*BMJ-British Medical Journal*	29
18	*Chest*	29

**Table 3:** Topmost global cited documents in the literature on tuberculosis.

Position	Paper	TCs	TC per Year
1	WHO 2012 Global Tuberculosis Report,	2485	276.1111
2 3	WHO Treatment of Tuberculosis: Guidelines, Fourth Edition, 2010,	634	57.6364
4	Moeller M, *Tuberculosis*,	151	13.7273
5	Machingaidze S, 2011, Pediatr Infect Dis J	124	12.4
6	Pooran A, 2013, PLoS ONE	115	14.375
7	Abd-El-Fattah Aa, 2013, Cell Biochem Biophys	98	12.25
8	Hanekom M, 2011, Tuberculosis	95	9.5
9	Muture Bn, 2011, Bmc Public Health	94	9.4
10	Ndjeka N, 2015, Int J Tuberc Lung Dis	88	14.6667
11	Moeller M, 2010, Fems Immunol Med Microbiol	86	7.8182
12	Starke Jr, 2014, Pediatrics	78	11.1429
13	Nhamoyebonde S, 2014, J Infect Dis	77	11
14	Schnippel K, 2018, Lancet Resp Med	76	25.3333
15	Friedrich So, 2011, J Clin Microbiol	72	7.2
16	Van Zyl L, 2015, Tuberculosis	71	11.8333
16	Marais Bj, 2010, Infect Dis Clin North Am	71	6.4545
17	Koegelenberg Cfn, 2010, Thorax	70	6.3636
18	Tabuti Jrs, 2010, J Ethnopharmacol	68	6.1818
19	Sutherland Js, 2010, J Immunol	66	6
20	Mesfin Ym, 2014, PLoS ONE	64	9.1429

**Table 4:** Topmost 21 relevant authors on tuberculosis outputs.

Position	Authors	Articles	Authors-Frac	Articles Fractionalized
1	[Anonymous]	133	[Anonymous]	133
2	Dheda K	44	Na Na	24
3	Walzl G	27	Burki T	15
4	Ameni G	25	Dheda K	13.4922
5	Na Na	24	Klein F	11
5	Van Helden PD	24	Passi GR	9
6	Goussard P	22	Nicol MP	8.7524
6	Warren RM	22	Cousins S	8
7	Nicol MP	21	Pillay M	7.8167
7	Pillay M	21	Loots DT	7.75
7	Schaaf HS	21	Mizrahi V	7.6667
8	Maartens G	19	Warner DF	7.3845
9	Mizrahi V	18	Manych M	7
9	Theron G	18	Goussard P	6.7286
10	Hatherill M	17	Ukwaja KN	6.1076
10	Tritar F	17	Ameni G	6.0129
10	Warner DF	17	Gorbach L	6
11	Hesseling AC	16	Gulland A	6
11	Ismail N	16	Kirby T	6
11	Loots DT	16	Zar HI	5.8786
11	Zar HJ	16	Marais BJ	5.6667

**Table 5:** Top 22 most relevant Africa countries by corresponding authors.

Position	Country	Articles	Freq	SCP	MCP	MCP_Ratio
1	South Africa	746	0.414444	717	29	0.03887
2	Ethiopia	223	0.123889	218	5	0.02242
3	Tunisia	153	0.085	152	1	0.00654
4	Morocco	131	0.072778	131	0	0
5	Nigeria	124	0.068889	122	2	0.01613
6	Egypt	95	0.052778	95	0	0
7	Uganda	48	0.026667	44	4	0.08333
8	Cameroon	42	0.023333	29	13	0.30952
9	Kenya	36	0.02	32	4	0.11111
10	Ghana	26	0.014444	22	4	0.15385
11	Benin	18	0.01	17	1	0.05556
12	Senegal	14	0.007778	14	0	0
12	Sudan	14	0.007778	13	1	0.07143
13	Tanzania	14	0.007778	13	1	0.07143
13	Algeria	13	0.007222	13	0	0
13	Congo	13	0.007222	11	2	0.15385
14	Zimbabwe	12	0.006667	8	4	0.33333
15	Madagascar	11	0.006111	10	1	0.09091
16	Togo	10	0.005556	8	2	0.2
17	Zambia	9	0.005	8	1	0.11111
18	Burkina Faso	8	0.004444	6	2	0.25
19	Malawi	6	0.003333	5	1	0.16667

**Table 6:** Top 20 most cited African countries in the tuberculosis literature.

Position	Country	Total Citations	Average Article Citations
1	South Africa	7816	10.477
2	Ethiopia	2125	9.529
3	Nigeria	555	4.476
4	Tunisia	514	3.359
5	Uganda	446	9.292
6	Egypt	366	3.853
7	Kenya	287	7.972
8	Cameroon	273	6.5
9	Morocco	204	1.557
10	Ghana	176	6.769
11	Gambia	140	28
12	Tanzania	84	6
13	Benin	77	4.278
14	Madagascar	50	4.545
15	Sudan	46	3.286
16	Zimbabwe	45	3.75
17	Zambia	34	3.778
18	Namibia	30	7.5
19	Malawi	28	4.667
20	Senegal	25	1.786

## Data Availability

Not applicable.

## Appendix A

**Table A1:** Analysis of authors impacts on tuberculosis publications.

Position	Author	h-Index	g-Index	m-Index	TC	NP	PY_Start
1	Dheda K	15	25	1.364	670	44	2010
2	Walzl G	11	19	1	378	27	2010
3	Ameni G	10	16	0.909	277	25	2010
4	Na Na	5	7	0.455	62	24	2010
5	Van Helden PD	9	19	0.9	386	24	2011
6	Goussard P	7	11	0.636	132	22	2010
7	Warren RM	9	21	0.9	456	22	2011
8	Nicol MP	10	15	0.909	243	21	2010
9	Pillay M	9	14	0.818	200	21	2010
10	Schaaf Hs	9	18	0.818	341	21	2010
11	Maartens G	6	16	0.6	261	19	2011
12	Mizrahi V	11	18	1	355	18	2010
13	Theron G	7	18	0.778	347	18	2012
14	Hatherill M	6	14	0.545	215	17	2010
15	Tritar F	2	2	0.286	7	17	2014
16	Warner DF	8	16	0.889	282	17	2012
17	Hesseling AC	7	14	0.7	208	16	2011
18	Ismail N	6	12	0.6	161	16	2011
19	Loots DT	9	15	1	251	16	2012
20	Zar HJ	8	15	0.8	250	16	2011
21	Ben Saad S	1	2	0.143	4	15	2014
22	Burki T	4	6	0.364	49	15	2010
23	Diacon AH	8	15	0.727	286	15	2010
24	Hoal EG	7	15	0.636	405	15	2010
25	Ukwaja KN	9	14	1	214	15	2012
